# An Experimental and Numerical Investigation on Enhancing the Ballistic Resistance of 316L Stainless Steel Plates Against Blunt Projectiles by Covering with 2024-T351 Aluminum Alloy Thin Plates

**DOI:** 10.3390/ma18184264

**Published:** 2025-09-11

**Authors:** Xinke Xiao, Qianqian Ma, Yifan Kong, Hao Lian, Jue Han, Yubo Gao

**Affiliations:** 1 School of Civil Engineering, Nanyang Institute of Technology, Nanyang 473004, China; 2 Henan International Joint Laboratory of Dynamics of Impact and Disaster of Engineering Structures, Nanyang Institute of Technology, Nanyang 473004, China; 3 School of Aerospace Engineering, North University of China, Taiyuan 030051, China; gaoyb@nuc.edu.cn

**Keywords:** 316L austenitic stainless steel, ballistic resistance, numerical simulation, bilayer target

## Abstract

To improve the ballistic resistance of hydrogen storage tank-grade 316L austenitic stainless steel (ASS) plates that are prone to shear plugging failure under blunt projectile impact, this study proposes a non-bonded bilayer protective configuration: covering the 316L ASS substrate with a thin front layer of 2024-T351 aluminum alloy (AA) plate. Ballistic impact tests were performed on monolithic 5 mm thick 316L ASS plates and bilayer targets composed of a 2.05 mm thick 2024-T351 AA plate and a 5 mm thick 316L ASS substrate (total thickness: 7.05 mm), using a single-stage light gas gun combined with high-speed photography. Parallel explicit dynamics models were established using ABAQUS/Explicit, incorporating a modified Johnson–Cook constitutive model and a Lode-dependent Modified Mohr–Coulomb (MMC) fracture criterion, thereby enabling rigorous mutual validation between experimental results and numerical simulations. Results demonstrate that the addition of a mere 2.05 mm thick aluminum alloy front layer significantly enhances the ballistic limit velocity (BLV) of the 5 mm thick 316L stainless steel target plate, increasing it from 167.5 m/s to 250.7 m/s. The enhancement mechanism is closely related to the transition in the failure mode from localized shear plugging to a combination of bulging, dishing, and plugging. This shift substantially improves the structure’s overall plastic deformation capacity and energy dissipation efficiency. This research provides an effective solution and establishes a reliable experimental–numerical benchmark for the lightweight, impact-resistant design of hydrogen storage tanks.

## 1. Introduction

Hydrogen energy serves as a crucial clean energy carrier that plays a pivotal role in advancing global carbon neutrality objectives [1,2,3]. The safety and efficiency of hydrogen storage are of paramount importance, with high-pressure vessels serving as the core infrastructure. Currently, 316L austenitic stainless steel (ASS) is widely recognized as the standard material for metallic hydrogen storage tanks due to its excellent resistance to both hydrogen embrittlement and corrosion [4,5]. However, the risk of catastrophic failure under accidental impact loads necessitates a comprehensive understanding of the ballistic impact behavior of 316L ASS in order to advance the safety design protocols for these critical components [6].

Compared to high-strength armor steels and low-density aluminum alloys, austenitic stainless steels (ASS) exhibit inferior ballistic resistance, which makes them less suitable for dedicated ballistic protection structures. As a result, research on the ballistic impact behavior of ASS plates remains limited [6]. Zhou [7] demonstrated that layered configurations of 316L stainless steel enhance energy absorption when subjected to blunt projectiles, whereas both monolithic and layered plates show similar resistance to ogival projectiles. Rodriguez-Martínez et al. [8] confirmed the superior energy dissipation capacity of 304 stainless steel compared to mild steel, which was attributed to strain-induced martensitic transformation. Jia et al. [9] systematically investigated the effects of temperature (−163 °C to 200 °C) on the ballistic resistance of 304 stainless steel, revealing that cryogenic conditions enhance material toughness due to phase transformation, with these findings supported by predictions from an advanced constitutive model. Dean et al. [10] reported a transition of failure modes in 304 stainless steel thin plates impacted by spherical projectiles: from dishing at 200 m/s to plugging at 300 m/s and fragmentation at 600 m/s. Zhao et al. [6] observed that 316L ASS plates experience shear plugging failure when impacted by blunt projectiles, with a relatively low ballistic limit velocity (BLV) of 180.9 m/s. In contrast, ogival projectiles induce ductile hole enlargement at a significantly higher BLV of 333.5 m/s. The consistently low ballistic resistance of monolithic 316 stainless steel under blunt projectile impact, primarily attributed to shear plugging failure, remains a fundamental challenge to the safety of hydrogen storage tanks. Overcoming this limitation through advanced material design, structural optimization, or effective control of failure mechanisms constitutes a key priority for future research.

Recent studies have demonstrated that multi-layer metallic systems can provide ballistic resistance that is comparable to, or even superior to, that of monolithic structures [11,12,13,14,15,16,17]. Specifically, according to our previous study [18], under the impact of blunt projectiles, transitioning the target plate structure from a monolithic to a double-layered configuration induces a fundamental shift in the dominant failure mechanism: shear plugging prevails in the former, whereas the latter is characterized by global bending and membrane stretching induced by bulging and dishing, which occur concurrently with plugging. The front plate typically fails through plugging, and the ejected plug subsequently impacts the second plate prior to the projectile. The sharp edges of the plug are rounded as a result of its forced interaction with the second plate, thereby altering the nature of the impact on the second plate—from that of a rigid, blunt-nosed projectile to a more deformable, hemispherical-like projectile. Consequently, plastic deformation becomes more spatially distributed and less confined to a narrow shear band. It is well known that the energy absorption capacity of shear plugging is significantly lower compared to that of global bending and membrane stretching induced by bulging and dishing.

Accordingly, to enhance the ballistic protective design of hydrogen storage tanks fabricated from 316L ASS while preserving a lightweight design to ensure mobility, the ballistic performance of a non-adhesively bonded bilayer system—comprising a 2024-T351 aluminum alloy (AA) front layer in direct contact with a 316L ASS plate—was investigated through a combined experimental–numerical approach. Ballistic tests were conducted using a single-stage light-gas gun to quantitatively evaluate and compare the ballistic performance of monolithic 5 mm thick 316L ASS targets with bilayer configurations consisting of a 2.05 mm thick 2024-T351 AA front layer in direct contact with a 5 mm thick 316L ASS plate. The primary objectives of these tests were to determine the BLVs and to characterize the failure modes exhibited by both target configurations. Transient dynamic responses and failure mechanisms within the 316L ASS plates were recorded using high-speed camera imaging and further analyzed through post-test structural examination. Concurrently, numerical simulations were performed using ABAQUS/Explicit (Version 2022), accurately replicating the experimental boundary conditions and target geometries. The computational models were rigorously validated against experimentally obtained BLVs and observed failure patterns. Subsequently, the validated models were utilized to investigate the underlying mechanisms responsible for the improved penetration resistance demonstrated by the bilayer configuration.

## 2. Ballistic Experiments

### 2.1. Test Setup

**Figure 1:**
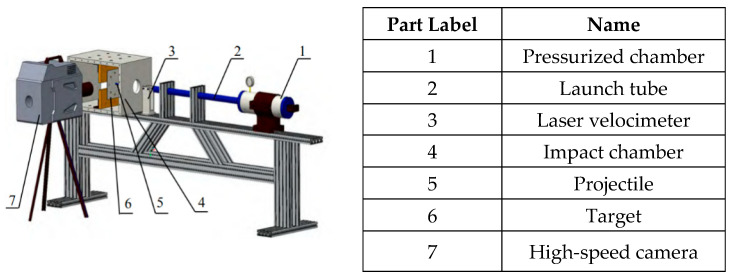
Illustration of the ballistic testing configuration.

The ballistic experiments were carried out employing a single-stage light gas gun, as depicted in Figure 1. The experimental setup included a high-pressure chamber, a launch tube, a device for measuring velocity, a target chamber, a projectile, and a high-speed camera system. During the test, pressurized nitrogen gas was utilized to drive the projectile along the launch tube. At the same time, a laser-based system measured the projectile’s initial velocity. Once the projectile entered the target chamber, the impact velocity, the development of penetration through the target plate, and the plate’s dynamic behavior were recorded using a high-speed imaging device (FASTCAMSA-Z, Photron Limited, Tokyo, Japan). This system operated at 60,000 frames per second, with each frame having an exposure time of 16.7 microseconds, thereby ensuring precise and detailed recording of the full impact process.

**Figure 2:**
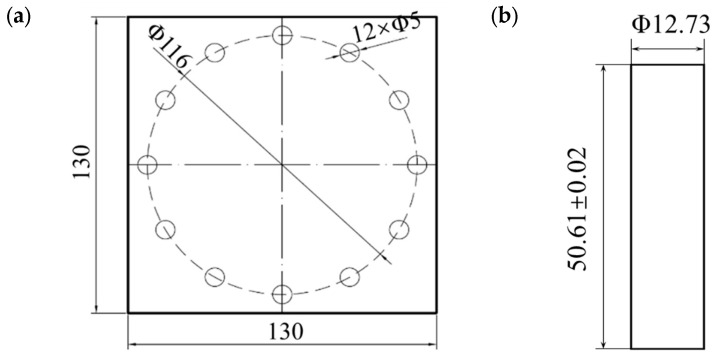
Geometry and measurements of the: (**a**) target, and (**b**) blunt projectile (unit: mm).

The monolithic plate was constructed from 316L ASS with a thickness of 5 mm. The 2024+316L bilayer target configuration consisted of a front plate fabricated from 2024-T351 AA with a thickness of 2.05 mm, and a rear plate composed of 316L ASS with an identical thickness of 5 mm. It should be noted that the 2024-T351 AA layer and the 316L ASS layer were mechanically stacked without any form of bonding or jointing. The projectile was manufactured from heat-treated, high-strength 38CrSi alloy steel, exhibiting a hardness of 52 HRC, a nominal diameter of 12.70 mm, and a nominal mass of approximately 50.60 g. Detailed geometric specifications for both the target plates and the projectile are presented in Figure 2. The target assembly was rigidly mounted to the support frame using eleven bolts, thereby enabling accurate monitoring and data acquisition during projectile impact, which provided a solid foundation for subsequent analysis.

### 2.2. Experimental Results

#### 2.2.1. Ballistic Limit Velocity

Ballistic impact experiments were performed on both monolithic 316L ASS targets and 2024+316L bilayer targets. The initial (*V*_0_) and residual velocities (*V*r) of projectiles are summarized in Table 1 and Table 2, along with their geometric specifications, including diameter (*d*_i_), length (*l*_i_), and mass (*m*). A residual velocity of zero indicates that the projectile either became stuck in the target plate or rebounded off it, whereas a residual velocity greater than zero represents the projectile’s velocity following complete perforation of the target.

For the monolithic 316L ASS targets, a total of nine impact tests were conducted, with initial velocities ranging from 153.5 m/s to 352.3 m/s. Similarly, fourteen impact experiments were carried out on the 2024+316L bilayer targets, covering an initial velocity range of 146.8 m/s to 322.2 m/s.

The Lambert–Jonas equation (L-J) [19] was employed to calculate the ballistic limit, as presented in Equation (1):

(1) $$V_{r}=a{\left(V_{0}^{p}-V_{\mathrm{bl}}^{p}\right)}^{1/p}$$

where *V*_bl_ represents the BLV of the target, and *a* and *p* are model parameters that control the curve’s shape. The model parameters and BLVs can be determined by applying the least squares fitting method to either experimentally or numerically obtained (*V*_i_, *V*r) datasets. It is important to emphasize that the L-J formula is an empirical equation, and the parameters *a* and *p* do not possess direct physical interpretations. From a mathematical standpoint, however, *a* represents the slope of the asymptote of the curve, while *p* functions as a shape factor that determines the sharpness with which the function approaches the asymptote. A detailed summary of the results from this analysis is provided in Table 3.

**Figure 3:**
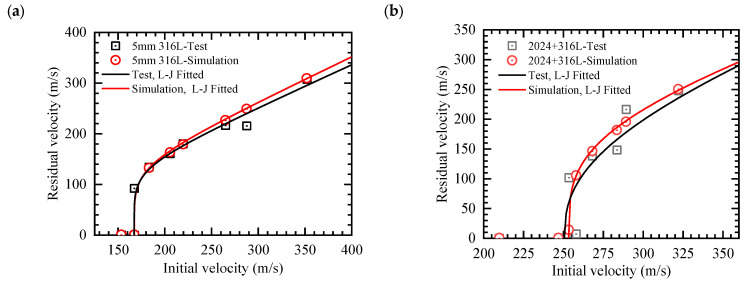
Correlation between projectile initial velocity and residual velocity: (**a**) 5 mm monolithic 316L ASS target, and (**b**) 2024+316L bilayer target.

The black curves in Figure 3, plotted using the L-J equation with the fitted parameters listed in Table 3, illustrates the relationship between initial and residual velocity. For the monolithic 5 mm thick 316L ASS target, the BLV was established at 167.3 m/s. Compared to this configuration, the 2024+316L bilayer targets demonstrated a substantially enhanced BLV of 250.7 m/s, corresponding to a 49.8% improvement in ballistic performance relative to the monolithic 316L ASS system. Moreover, compared to the previously reported BLV of 180.9 m/s for a 10 mm thick monolithic 316L ASS plate [6], the 2024+316L bilayer target exhibits a 38.5% improvement in ballistic performance. Notably, this significant enhancement in impact resistance was achieved without any corresponding increase in total areal density.

#### 2.2.2. Failure Mechanism of the Targets

**Figure 4:**
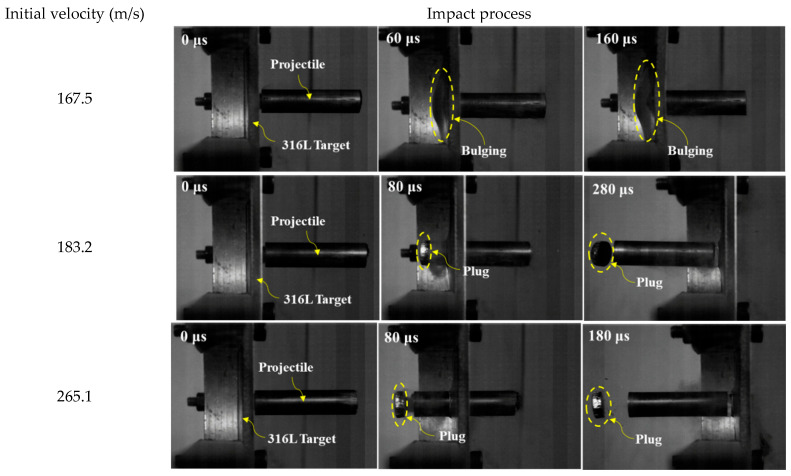
Selected high-speed imaging frames captured at different impact velocities for the monolithic 316L targets.

**Figure 5:**
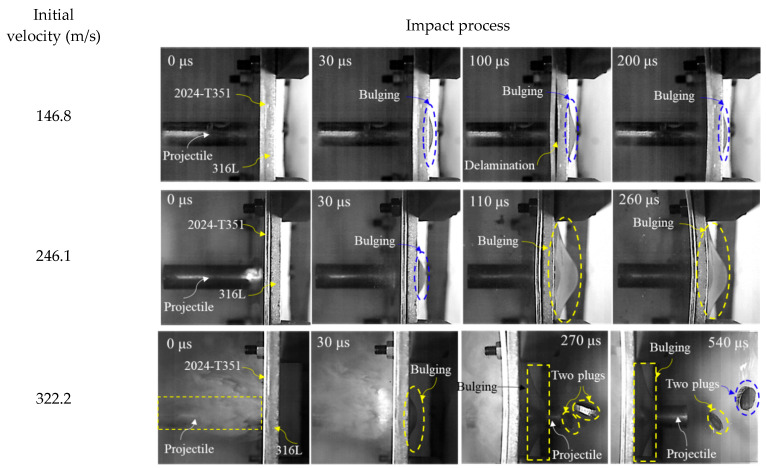
Selected high-speed imaging frames captured at different impact velocities for 2024+316L bilayer targets.

**Figure 6:**
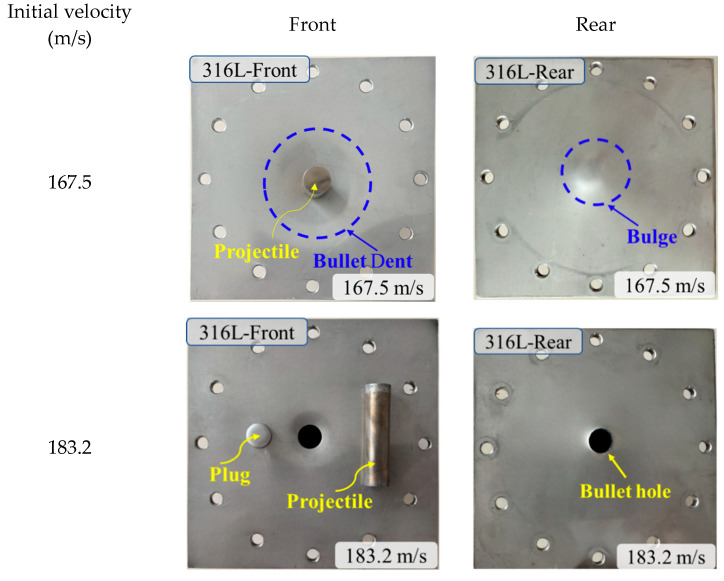

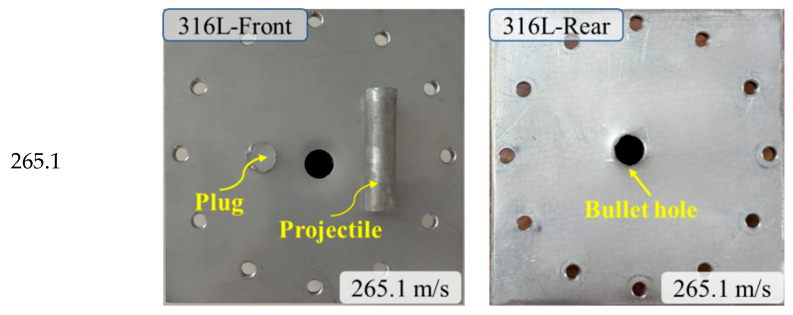
Fracture patterns of the monolithic 316L ASS targets.

**Figure 7:**
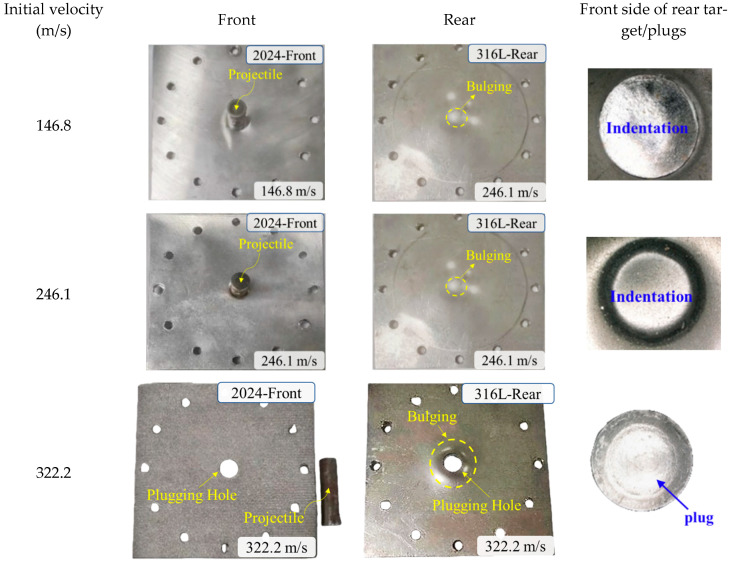
Fracture patterns of the 2024+316L bilayer targets.

To investigate the failure mechanisms resulting from blunt projectile impacts on the target materials, the impact events were captured using a high-speed camera. Representative experimental images acquired at three distinct impact velocity regimes—subcritical, near-critical, and supercritical relative to the ballistic limit velocity (BLV)—are displayed in Figure 4 and Figure 5. The corresponding recovered targets, plug formations, and projectiles for these impacts are shown in Figure 6 and Figure 7.

Observations confirm that all projectiles struck the targets at normal incidence. For the monolithic 316L ASS target, perforation consistently occurred through shear plugging, resulting in the ejection of a plug whose diameter was marginally less than that of the projectile. This failure mode was accompanied by permanent target deformation, the extent of which diminished as the impact velocity increased.

The failure response of the 2024+316L bilayer target exhibited significant differences compared to the monolithic configuration. The front 2024-T351 AA layer predominantly failed through shear plugging. In contrast, the rear 316L ASS layer displayed a composite failure mode characterized by bulging, dishing, and plugging. At impact velocities below the BLV—for instance, *V*_0_ = 146.8 m/s and V_0_ = 246.1 m/s—the projectile penetrated the front layer, ejecting a plug that subsequently impacted the rear 316L ASS target. The combined impact of the projectile and the plug ejected from the front AA plate resulted in both indentation and localized bulging on the rear 316 ASS plate. At velocities slightly exceeding the BLV—for example, *V*_0_ = 322.2 m/s—complete perforation occurred, yielding two distinct plugs. In this velocity regime, the rear 316L ASS plate demonstrated markedly greater deformation compared to the front 2024-T351 AA plate.

To fundamentally elucidate the velocity-dependent failure transition in the layered target, the distinct failure mechanisms governing each layer must be decoupled. High-speed imaging and post-mortem analysis (Figure 7) demonstrate that perforation of the front 2024-T351 AA layer is governed by shear plugging, resulting in clean cylindrical plug ejection with minimal peripheral deformation. In contrast, the rear 316L ASS layer undergoes a sequential hybrid failure mechanism: initial global biaxial tensile stretching and bulging induced by impact stress waves, followed by progressive shear localization. This localization culminates in plug ejection. The detached 316L plugs, exhibiting shear lips and microscopic dimpled rupture surfaces visible in Figure 7, provide direct evidence of this multi-stage ductile failure process.

Therefore, these analyses demonstrate that the introduction of the 2024-T351 AA front layer fundamentally alters the deformation and failure behavior of the rear 316L ASS plate under blunt projectile impact. Unlike failure through localized shear plugging as observed in the monolithic configuration, the rear steel layer undergoes substantial energy absorption via large-scale global bending and membrane stretching inducted by bulging and dishing prior to final plug formation and ejection. This transition to a multi-stage, ductile hybrid failure mode constitutes the primary mechanism responsible for the enhanced ballistic resistance exhibited by the 2024+316L bilayer target plate.

## 3. Numerical Simulation

### 3.1. Finite Element Model

**Figure 8:**
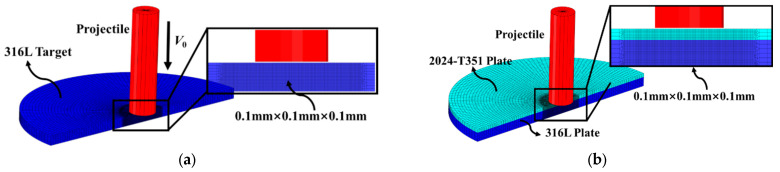
Finite element model of the ballistic tests: (**a**) monolithic target, and (**b**) bilayer target.

Within the ABAQUS/Explicit finite element framework—a Dassault Systèmes module specialized in transient nonlinear dynamic simulations—a symmetric three-dimensional model was developed to simulate the impact event, as illustrated in Figure 8. Due to its significantly greater stiffness and minimal plastic deformation relative to the target, the projectile was modeled as a rigid body to improve computational efficiency. A localized region of interest—defined by a 10 mm diameter circle centered on the target plate—was selected for refined meshing. To accurately reproduce the complex deformation and dynamic response in this critical zone, a fine uniform grid dimension of 0.1 mm × 0.1 mm × 0.1 mm was utilized, in line with established methodologies cited in [18,20,21]. The simulation employed C3D8R hexahedral elements. To simulate crack initiation and propagation induced by the impact, the element deletion technique was implemented. Contact interactions—encompassing projectile-target and inter-plate interfaces—were modeled via a general contact algorithm with “Hard Contact” normal behavior and frictionless tangential formulation. Target plate boundaries were fully constrained by eliminating all translational degrees of freedom.

### 3.2. Material Models

To accurately capture the plasticity of 316L ASS and 2024-T351 AA under ballistic impact loading, which involves large strains, high strain rates, and temperature transients, this study employs the phenomenological Modified Johnson–Cook (MJC) plasticity model previously used in [17]. This model builds upon the original Johnson–Cook (JC) framework [22], which is widely used in impact dynamics for its simplicity. Critically, the MJC model overcomes a key limitation of the JC model by refining its strain hardening description through a linear combination of Ludwik and Voce laws [23,24,25]. Furthermore, compared to the original JC model, the MJC model introduces an additional parameter *p*, enabling greater flexibility in describing the nonlinear relationship between material flow stress and temperature elevation. Similar to its predecessor, the MJC formulation decouples strain hardening, strain rate hardening, and thermal softening into three multiplicative components. The constitutive equation is expressed as

(2) $$\sigma_{\mathrm{eq}}=\left\{\alpha (A+B\varepsilon_{\mathrm{eq}}^{n})+(1-\alpha )\left[A+Q(1-e^{-\beta \varepsilon_{\mathrm{eq}}})\right]\right\}(1+C\ln{\dot{\varepsilon }}_{\mathrm{eq}}^{*})(1-pT^{*m})$$

where σ_eq_ is the von Mises stress; $\varepsilon_{\mathrm{eq}}$ is the equivalent plastic strain; ${\dot{\varepsilon }}_{\mathrm{eq}}^{*}$ is the dimensionless strain rate given by ${\dot{\varepsilon }}_{\mathrm{eq}}^{*}={\dot{\varepsilon }}_{\mathrm{eq}}/{\dot{\varepsilon }}_{o}$, where ${\dot{\varepsilon }}_{o}$ and ${\dot{\varepsilon }}_{\mathrm{eq}}$ are the present strain rate and reference strain rate, respectively; and T* is the homologous temperature defined by $T^{*}=(T-T_{0})/(T_{m}-T_{0})$, where *T* is the actual temperature, *T*_0_ is the room temperature, and *T*_m_ is the melting temperature of the material. *α*, *A*, *B*, *n*, *Q*, *β*, *C*, and *m* are material constants to be determined.

Substantial research has demonstrated that incorporating the Lode parameter into fracture criteria can significantly improve numerical prediction accuracy for ductile failure [26,27,28,29,30,31,32]. Consequently, this study adopts the Lode-dependent Modified Mohr–Coulomb (MMC) fracture criterion [27] to characterize the fracture behavior of 316L ASS and 2024-T351 AA. The MMC fracture criterion reads

(3) $$\begin{array}{l} \varepsilon_{f}={\left(\frac{K}{c_{2}}\left[c_{\theta }^{s}+\frac{\sqrt{3}}{2-\sqrt{3}}(c_{\theta }^{\alpha x}-c_{\theta }^{s})(\sec(\frac{\bar{\theta }\pi }{6})-1)\right]\left[\sqrt{\frac{1+c_{1}^{2}}{3}}\cos(\frac{\bar{\theta }\pi }{6})+c_{1}(\eta +\frac{1}{3}\sin(\frac{\bar{\theta }\pi }{6}))\right]\right)}^{-\frac{1}{n}}\\ \;\;\;\;\;(1+D_{4}\ln{\dot{\varepsilon }}_{\mathrm{eq}}^{*})(1+D_{5}T^{*D_{6}}) \end{array}$$

(4) $$c_{\theta }^{\alpha x}=\left\{\begin{array}{c} 1\\ c_{\theta }^{c} \end{array}\right.\begin{array}{c} \mathrm{for}\;\bar{\theta }\geq 0\\ \mathrm{for}\;\bar{\theta }<0 \end{array},$$

(5) $$\bar{\theta }=1-\frac{2}{\pi }\mathrm{arc}\cos[\frac{13.5}{\sigma_{\mathrm{eq}}^{3}}(\sigma_{1}-\sigma_{m})(\sigma_{2}-\sigma_{m})(\sigma_{3}-\sigma_{m})],$$

(6) $$\sigma_{m}=\frac{1}{3}(\sigma_{1}+\sigma_{2}+\sigma_{3}),$$

In the aforementioned Equations (3)–(6), $\varepsilon_{f}$ is the fracture strain, *D*_4_–*D*_6_ are the model constants, *η* is the stress triaxiality defined by *η* = (*σ*_1_ + *σ*_2_ + *σ*_3_)/(3*σ*_eq_), where *σ*_1_, *σ*_2_, and *σ*_3_ are the maximum, intermediate, and minimum principal stress, respectively. $\bar{\theta }$ is the normalized Lode angle parameter; *σ*_m_ is the mean normal stress. *K*, *n*, $c_{\theta }^{s}$, $c_{\theta }^{c}$, $c_{1}$ and $c_{2}$ are the model parameters of the material.

Given the transient impact dynamics, adiabatic heating is modeled via the thermal conversion integral:

(7) $$\Delta T=\frac{\chi }{\rho C_{P}}\int \sigma_{\mathrm{eq}}d\varepsilon_{\mathrm{eq}}$$

where *ρ* denotes material density, *C*_p_ is the specific heat, and χ is the Taylor–Quinney coefficient quantifying the conversion fraction of plastic work to heat. Based on empirical evidence for metallic materials under high strain rate conditions [33,34], a value of χ = 0.9 is adopted in this study.

To simulate fracture in target plates under ballistic impact, a damage-initiated element erosion approach was employed. This technique removes elements from the computation when their damage accumulation variable reaches unity (*D* = 1). The linear damage evolution law is defined as

(8) $$D=\sum \Delta \varepsilon_{\mathrm{eq}}/\varepsilon_{f}$$

where $\varepsilon_{f}$ denotes the fracture strain at current status, and $\Delta \varepsilon_{\mathrm{eq}}$ represents the equivalent plastic strain increment during an explicit integration increment.

As detailed in our prior studies [6,31], the constitutive and fracture model parameters for both materials were primarily calibrated through mechanical testing coupled with inverse finite element analysis. Experimental methodologies, resulting data, and parameter optimization procedures are comprehensively documented therein. Accordingly, the final material parameters for 316L austenitic stainless steel (ASS) and 2024-T351 aluminum alloy (AA) are summarized in Table 4 of the present study.

The material modeling parameters of 2024-T351 AA were derived from [31], and those for 316L ASS were initially derived from the work of Zhao et al. [6]. However, preliminary simulations using these parameters exhibited substantial deviations from experimental observations. To address this deviation, the strain rate sensitivity parameter *C* was systematically optimized through iterative computations guided by experimentally obtained BLVs of the monolithic 316L ASS target. This calibrated parameter not only improved the alignment between numerical predictions and experimental results but also preserved the constitutive framework of the original model. Subsequent validation confirmed that the adjustment enhanced predictive accuracy while maintaining consistency with the underlying physical assumptions of the reference material model.

## 4. Numerical Findings and Their Comparison to Experimental Data

This section presents the numerical simulation results related to the ballistic limit velocity and failure modes of the target. The reliability of the numerical model is confirmed by comparison with experimental observations. Based on the validated simulation results, the underlying mechanisms contributing to the improved protective performance of the bilayer target compared to the monolithic target are further analyzed.

### 4.1. Ballistic Limit Velocities

The initial and residual velocity data derived from numerical simulations are compiled in Table 5 and illustrated in Figure 3. By employing the L-J equation (Equation (1)) and utilizing the least squares fitting technique, the ballistic limit curves and their associated BLV values were calculated. These outcomes are depicted in Figure 3 and summarized in Table 3.

The analysis indicates a strong agreement between simulation outcomes and experimental data. For the 5 mm monolithic 316L ASS target, the simulated BLV of 167.3 m/s closely matches the experimentally obtained value of 167.5 m/s. In the case of the 2024+316L bilayer target, the simulated BLV of 253.4 m/s exhibits a deviation of only 1.1% from the experimental value of 250.7 m/s.

These findings demonstrate that the numerical model utilized in this study accurately captures the ballistic limit behavior of both monolithic 316L plates and 2024+316L bilayer targets under impact from blunt projectiles.

### 4.2. Failure Pattern

This section evaluates the predictive capability of the numerical model in capturing structural damage by comparing the failure modes observed in simulations with those from experimental results. The analysis covered three distinct impact velocity regimes: velocities slightly below, slightly above, and well above the BLV.

**Figure 9:**
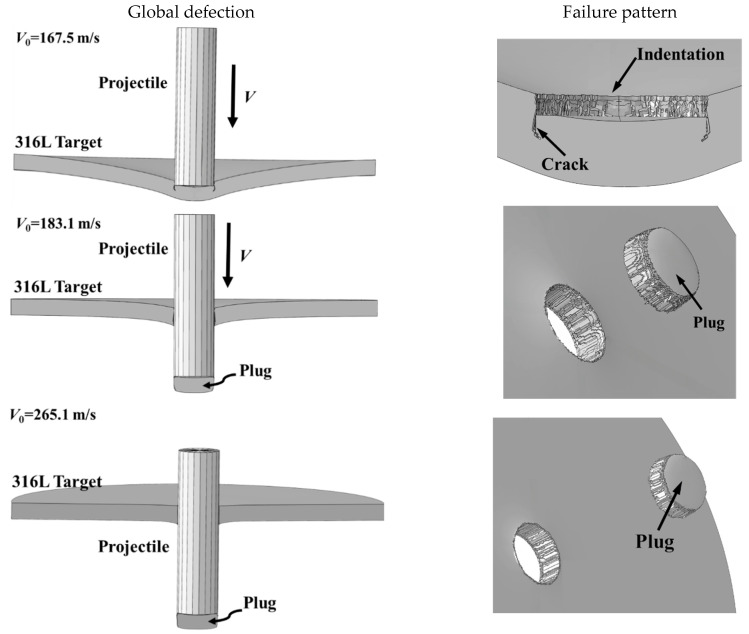
Predicted failure patterns of the monolithic targets.

Figure 9 depicts the predicted failure behavior of the 316L AAS monolithic targets. As observed, the simulations effectively captured all key failure characteristics, demonstrating a high level of accuracy. The simulation results exhibited a strong match with the experimental findings shown in Figure 4 and Figure 6.

The target consistently exhibited characteristic shear plugging failure. Under impact velocities below the ballistic BLV, a circular indentation approximately equal in diameter to the projectile was formed on the front face, surrounded by circumferential cracks. Concurrently, significant global deflection occurred in the central region of the target. When the impact velocity exceeded the BLV, perforation occurred through the ejection of a cylindrical plug, resulting in the formation of a clean hole free of fragments. Moreover, the magnitude of global deflection decreased progressively with increasing impact velocity.

**Figure 10:**
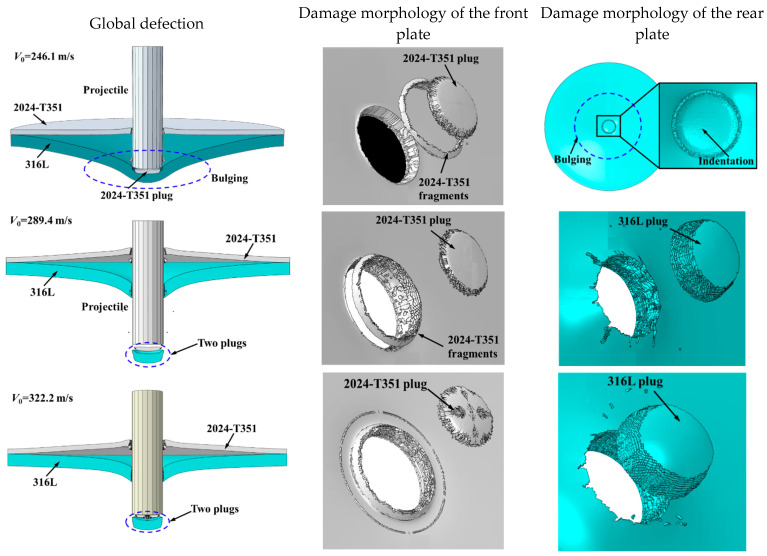
Predicted failure patterns of the 2024+316L bilayer targets.

Figure 10 presents the predicted damage morphology of the bilayer 2024+316L targets. As seen, the failure behavior exhibited significant variation between the two plates. The first plate experienced dominant shear plugging failure with minimal global deformation. The ejected plug from the first plate reduced the occurrence of intense shear localization in the second plate. Consequently, the second plate primarily failed through global bending, membrane stretching, and finally plugging. The simulations accurately predicted these experimentally observed fracture characteristics. Specifically, the simulations captured the ejection of distinct cylindrical plugs from both the first and second plates, as well as the formation of small petalling fractures on the bulged surface of the rear plate. The numerical predictions of the failure mode for the plates showed excellent consistency with the experimental findings shown in Figure 5 and Figure 7.

In summary, the comparative analysis of failure modes in both monolithic and bilayer targets under varying impact velocities highlights the robust predictive capability of the numerical model based on the MMC fracture criterion. The simulations accurately captured the characteristic shear plugging failure in monolithic targets, including the transition from indentation and global deflection below the BLV to clean perforation accompanied by reduced deflection above it. For bilayer configurations, the model successfully replicated the distinct failure mechanisms: shear plugging in the front plate, and bulging, dishing, and plugging in the rear plate. The close agreement between numerical predictions and experimental observations across all investigated cases confirms the model’s effectiveness in simulating structural damage under ballistic impact.

## 5. Enhancement Mechanism of 2024-T351 AA Front Layer on the Ballistic Performance of 316L ASS Target

Experimental and numerical results demonstrate that adding a 2.05 mm thick layer of 2024-T351 AA in front of a 5 mm thick 316L AAS target significantly enhances the BLV of the system, increasing it from 167.5 m/s for the monolithic 316L ASS target to 250.7 m/s for the bilayer configuration. This section presents a qualitative analysis of the underlying mechanisms based on experimental and simulation observations, along with quantitative validation through numerical simulations conducted at selected impact velocities.

### 5.1. Energy Absorption Fundamentals and Core Dissipation Mechanisms

**Figure 11:**
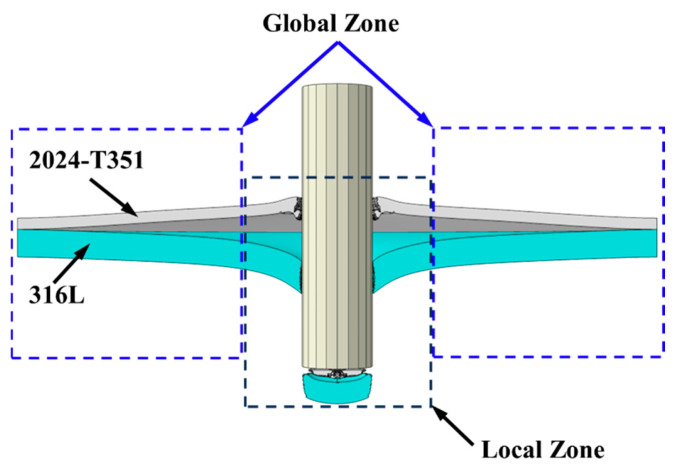
Definition of local and global deformation zones.

A schematic view of local and global deformation zones is shown in Figure 11. When the impact velocity reaches or exceeds the BLV, a portion of the projectile’s kinetic energy is transferred to the target system. The total energy absorbed by the impact can be expressed using the following equation [35]:

(9) $$\Delta W=W_{E}+W_{P}^{\mathrm{Local}}+W_{P}^{\mathrm{Global}}+W_{F}+W_{K}^{\mathrm{Plug}}=\frac{1}{2}m(V_{0}^{2}-V_{r}^{2})$$

where $W_{E}$ is the elastic strain energy, $W_{P}^{\mathrm{Local}}$ represents the energy dissipated by localized plastic deformation (primarily shear plugging), $W_{P}^{\mathrm{Global}}$ denotes the energy dissipated by global plastic deformation (primarily bending), $W_{F}$ corresponds to the energy dissipated by friction, and $W_{K}^{\mathrm{Plug}}$ is the kinetic energy imparted to the plug.

According to the literature [36,37], *W*_E_ typically accounts for less than 3% of the total energy, and *W*_F_ for less than 5%. Consequently, these terms can be neglected in the energy balance. Thus, the core energy dissipation mechanisms during impact are concentrated in Equation (10):

(10) $$\Delta W=W_{P}^{\mathrm{Local}}+W_{P}^{\mathrm{Global}}+W_{K}^{\mathrm{Plug}}$$

### 5.2. Enhancement Mechanism of the 2024-T351 AA Layer

Analysis of experimental and numerical results reveals that the introduction of the 2024-T351 aluminum alloy front layer substantially enhances the ballistic resistance of the 316L steel target through two primary mechanisms:

#### 5.2.1. Energy Absorption by the Front Layer

The 2.05 mm thick 2024-T351 AA layer absorbs a portion of the projectile’s initial kinetic energy primarily through localized shear plugging failure, accompanied by limited plastic deformation.

#### 5.2.2. Transformation of the Failure Mechanism and Energy Absorption in the Main 316L ASS Plate

The presence of the 2024-T351 AA layer fundamentally alters the failure mode and energy absorption characteristics of the rear 316L steel target. The monolithic 5 mm 316L steel target fails predominantly through localized shear plugging under projectile impact. In the bilayer structure, the front AA layer fails mainly by shear plugging. Crucially, the failure mode of the rear 316L main target transitions into a synergistic combination of bulging, dishing, and plugging, predominantly governed by bending and stretching deformation mechanisms.

A key factor contributing to the enhanced energy absorption capacity of the bilayer structure is the significant increase in global plastic work, *W*_P_^Global^. For the monolithic 316L target impacted by a blunt projectile, plastic deformation is highly concentrated in the shear plugging path, with minimal deformation elsewhere. In contrast, within the bilayer system, the plug ejected from the front aluminum layer impacts the rear 316L plate prior to the projectile. As previously noted, the sharp edges of the plug originating from the front AA plate become rounded due to its forced interaction with the rear 316L ASS plate, thereby altering the impact characteristics on the latter. Consequently, plastic deformation resembling that induced by a hemispherical projectile develops, rather than shear plugging. It should be noted that, under impact by a hemispherical projectile—particularly at velocities near the BLV—a ductile metallic plate typically exhibits pronounced local (indentation-induced) and global (bulging-related) plastic deformation. Consequently, the bilayer structure absorbs significantly more energy through this markedly increased W_P_^Global^.

### 5.3. Numerical Validation of the Enhancement Mechanism

To quantitatively validate the proposed energy dissipation mechanisms, numerical simulations were conducted on both the monolithic 316L stainless steel target and the 2024-T351 aluminum alloy + 316L ASS bilayer target. Impacts were simulated at 1.06 times their respective BLVs, corresponding to impact velocities of 177.6 m/s for the monolithic configuration and 265.7 m/s for the bilayer configuration. This velocity range ensures complete penetration while allowing sufficient development of plastic deformation, thereby providing appropriate conditions for analyzing the energy dissipation mechanisms. To enable a direct comparison of protective performance under equivalent impact energy, the monolithic 316L ASS target was also subjected to impacts at the bilayer target’s velocity of 265.7 m/s. Plastic energy dissipation data for local (*W*_P_^Local^) and global (*W*_P_^Global^) deformation zones were extracted from the simulation results, with a focus on quantifying and comparing their distribution characteristics, as summarized in Table 6.

#### 5.3.1. Energy Response of Monolithic 316L ASS Target

At 177.6 m/s, energy dissipation in the monolithic 316L target is predominantly attributed to WPLocal, amounting to 137.5 J (34.5% of the total system energy). In contrast, W_P_^Global^ accounts for only 45.84 J (11.5%). This supports the conclusion that near the BLV, energy dissipation primarily occurs through the localized shear plugging mechanism within the projectile contact region, with minimal contribution from global deformation.

When the impact velocity increases to 265.7 m/s—corresponding to the same projectile kinetic energy as in the bilayer simulation—the absolute value of *W*_P_^Local^ rises to 157.7 J, yet its relative contribution drops significantly to 17.6%. More importantly, *W*_P_^Global^ decreases sharply to only 12.6 J, representing a mere 1.4% of the total energy. This underscores a fundamental limitation of the monolithic structure: as impact energy increases substantially, plastic deformation becomes increasingly localized, the efficiency of global deformation in dissipating energy effectively disappears, and failure remains confined to the relatively inefficient shear plugging mechanism.

#### 5.3.2. Energy Distribution and Interlayer Interaction Mechanism in Bilayer Targets

Under identical impact conditions at 265.7 m/s (projectile kinetic energy: 893.1 J), the bilayer structure demonstrates a markedly different energy dissipation profile compared to the monolithic target.

The 2024-T351 AA front plate primarily dissipated energy through the formation of localized shear plugging, absorbing 98.6 J (11.0% of the total impact energy) via *W*_P_^Local^. Its *W*_P_^Global^ contribution was negligible at 1.43 J (0.16%), consistent with its role as a sacrificial energy absorber. The core mechanism involves the transfer of kinetic energy through high-velocity plug ejection during the initial projectile engagement.

The 316L ASS main target demonstrates a *W*_P_^Local^ of 262.8 J (29.4% of total energy), which is substantially higher than that of the front plate. More notably, its W_P_^Global^ increases to 175.8 J (19.7% of the total energy). When compared to the monolithic 316L target under the same kinetic energy impact (*W*_P_^Global^ = 12.6 J), the global energy dissipation in the rear plate is nearly 13 times greater. Moreover, its contribution ratio (19.7%) markedly exceeds the efficiency achieved by the monolithic target under equivalent conditions (only 1.4%).

The secondary impact of the high-velocity plug generated by the front plate onto the rear plate, combined with the mechanical constraints resulting from interlayer interaction, synergistically induces extensive global plastic deformation in the rear plate. This alters the failure mode of the rear plate from the monolithic target’s single shear plugging mechanism to a composite failure mode involving both bending deformation and plugging. The substantial increase in the rear plate’s *W*_P_^Global^ accounts for 18.3% of the total projectile energy, quantitatively validating the critical role of interlayer interaction in activating global plastic deformation within the rear plate.

#### 5.3.3. Mechanical Essence of Ballistic Performance Enhancement

The bilayer structure induces a qualitative leap in energy dissipation efficiency.

Total Plastic Dissipation: The proportion of total plastic energy dissipation (*W*_P_^Local^ + *W*_P_^Global^) in the bilayer structure reaches 71.4% (front plate: 11.16%; rear plate: 60.24%). This corresponds to a 276% increase compared to the 19.0% observed for the monolithic target under the same impact energy.

Global Plastic Dissipation: The total contribution of *W*_P_^Global^ to the system energy increases significantly from 1.4% in the monolithic target to 31.2% in the bilayer structure (front plate: 0.16%; rear plate: 19.7%; synergistic contribution: remaining portion).

Failure Mode Transformation: The energy dissipation ratio *W*_P_^Global^/*W*_P_^Local^ for the rear plate reaches 0.67, significantly higher than the ratio of 0.08 for the monolithic target. This indicates a transition of the rear plate to a highly efficient energy dissipation mode, where bending deformation assumes a dominant and synergistic role.

In summary, the AA front plate effectively reconstructs the failure mechanism of the 316L main target through both the kinetic energy transfer associated with plug generation and the imposition of mechanical interlayer constraints. This reconstruction transfers the failure mode from an inefficient localized shear plugging mechanism (*W*_P_^Global^ ≤ 1.4%) to an efficient synergistic mechanism involving bulging and dishing (*W*_P_^Global^ = 19.7%). As a result, the system’s overall energy absorption efficiency increases by nearly threefold, and the contribution of global plastic energy dissipation is enhanced by over thirteen times. These findings provide solid numerical evidence and a clear mechanical explanation for optimizing energy dissipation pathways through interlayer coupling effects in composite armor design, thereby enabling significant performance improvements, such as the 49.7% increase in BLV.

## 6. Conclusions

This study employed a hybrid experimental–numerical approach to investigate the ballistic resistance of bilayer 316L austenitic stainless steel targets reinforced with a 2024-T351 aluminum alloy front layer against blunt projectiles. Ballistic impact tests were carried out using a single-stage gas gun on both monolithic 5 mm thick 316L ASS targets and bilayer 2024+316L configurations, consisting of a 2.05 mm thick 2024-T351 aluminum alloy front layer in direct contact with a 5 mm thick 316L rear plate. Parallel finite element models were constructed, and simulations were conducted using ABAQUS/Explicit. The simulated ballistic limit velocities and failure modes were rigorously validated against experimental data. Based on the combined experimental and numerical findings, the enhancement mechanism of the 2024-T351 aluminum alloy front layer on the ballistic performance of the 316L steel plate was systematically analyzed. The key conclusions are summarized as follows:

(1) The incorporation of a 2.05 mm thick 2024-T351 aluminum alloy front layer significantly increased the ballistic limit velocity of the 5 mm thick 316L steel target by 49.7%, from 167.5 m/s to 250.7 m/s.

(2) The aluminum alloy front layer reconfigured the failure mechanism of the rear 316L main plate through kinetic energy transfer from the generated plug and the imposition of interlayer constraints. Whereas monolithic 316L austenitic stainless steel (ASS) targets failed predominantly via localized shear plugging, the rear 316L plate in the bilayer configuration transitioned to a more efficient bulging, dishing, and plugging mode.

(3) The bilayer structure demonstrated a substantial improvement in both total plastic energy dissipation (*W*_P_^Local^ + *W*_P_^Global^) and global plastic energy dissipation (W_P_^Global^) efficiency.

In summary, the fundamental mechanism responsible for the 49.7% improvement in the ballistic limit velocity of the bilayer structure can be attributed to the 2024-T351 aluminum alloy front layer. This layer enables a transition in the failure mode of the rear steel plate, shifting from inefficient localized shear plugging to a highly efficient synergistic mechanism involving bulging, dishing, and plugging, while significantly improving the global plastic energy absorption capacity. The study offers robust experimental and numerical evidence, supported by a clear mechanical interpretation, for leveraging interlayer coupling effects to optimize energy dissipation pathways and improve protective performance in composite armor design for hydrogen storage tanks.

It should be acknowledged that the present study has certain limitations. Specifically, the investigation did not comprehensively explore the influence of bonding conditions between the 316L stainless steel plate and the thin aluminum layer, nor did it systematically examine the effects of different interlayer materials. Additionally, variables such as the shape, dimensions, and material of the projectiles, the incident angles and distances of fire, the thickness of the individual plates, and the microstructural characteristics of both materials were not fully addressed, which may restrict the generalizability of the findings regarding the anti-penetration performance enhancement of 316L stainless steel.

## Figures and Tables

**Table 1:** Ballistic impact test results of monolithic 316L austenitic stainless steel targets subjected to impacts from blunt projectiles.

NO.	Initial Diameter *d*_i_ (mm)	Initial Length *l*_i_ (mm)	Initial Mass *m* (g)	Initial Velocity *V*_0_ (m/s)	Residual Velocity *V*r (m/s)	Status
1	12.66	50.60	49.52	287.8	215.4	Perforated
2	12.66	50.61	49.53	265.1	216.4	Perforated
3	12.66	50.57	49.55	167.4	0	Projectile got stuck
4	12.66	50.50	49.55	219.7	180.1	Perforated
5	12.66	50.55	49.55	183.2	134.1	Perforated
6	12.66	50.56	49.53	205.8	160.8	Perforated
7	12.66	50.82	49.55	153.5	0	Projectile got stuck
8	12.66	50.60	49.55	167.5	92.2	Perforated
9	12.66	50.61	49.56	352.3	307.1	Perforated

**Table 2:** Ballistic impact test results of 2024+316L bi-layer target subjected to impacts from blunt projectiles.

NO.	Initial Diameter *d*_i_ (mm)	Initial Length *l*_i_ (mm)	Initial Mass *m* (g)	Initial Velocity *V*_0_ (m/s)	Residual Velocity *V*r (m/s)	Status
1	12.72	50.61	50.27	246.1	0.00	Projectile got stuck
2	12.73	50.60	50.32	146.8	0.00	Projectile got stuck
3	12.72	50.56	50.23	250.7	0.00	Projectile got stuck
4	12.73	50.49	50.16	246.9	0.00	Projectile got stuck
5	12.73	50.59	50.27	268.2	138.5	Perforated
6	12.73	50.58	50.31	209.8	0.00	Projectile got stuck
7	12.75	50.83	50.30	246.7	0.00	Projectile got stuck
8	12.73	50.62	50.29	283.6	148.3	Perforated
9	12.73	50.62	50.32	289.4	216.3	Perforated
10	12.72	50.61	50.33	246.7	0.00	Projectile got stuck
11	12.74	50.60	50.31	247.2	0.00	Projectile got stuck
12	12.74	50.59	50.28	258.1	7.38	Perforated
13	12.72	50.64	50.29	253.5	101.8	Perforated
14	12.73	50.66	50.30	322.2	248.0	Perforated

**Table 3:** Constants of Lambert–Jonas equation.

Target	Test			Simulation		
	*a*	*p*	*V*_bl_ (m/s)	*a*	*p*	*V*_bl_ (m/s)
316L monolithic	0.84	5.35	167.3	0.88	4.95	167.5
2024+316L bilayer	0.99	2.50	250.7	0.91	3.44	253.4

**Table 4:** Material constants of 316L ASS [6] and 2024-T351 AA [31].

316L ASS			2024-T351 AA		
Description	Notation	value	Description	Notation	value
Modulus of elasticity	*E* (GPa)	183.3	Modulus of elasticity	*E* (GPa)	72.0
Poisson’s ratio	*v*	0.3	Poisson’s ratio	*v*	0.3
Density	*ρ* (kg/m^3^)	7900	Density	*ρ* (kg/m^3^)	2770
Yield stress constant	*A* (MPa)	266.2	Yield stress constant	*A* (MPa)	360.3
Strain hardening constant	*B* (MPa)	1263.37	Strain hardening constant	*B* (MPa)	649.4
	*n*	0.72		*n*	0.68
	*Q* (MPa)	1068.6		*Q* (MPa)	235.9
	*β*	2.373		*β*	8.988
	*α*	0.51		*α*	0.104
Strain rate constant	*C*	0.025	Strain rate constant	*C*	0.0146
Thermal softening constant 1	*p*	1.0	Thermal softening constant 1	*p*	1.702
Thermal softening constant 2	*m*	0.8	Thermal softening constant 2	*m*	2.768
Reference strain rate	${\dot{\varepsilon }}_{0}\;(/s)$	8.33 × 10^−4^	Reference strain rate	${\dot{\varepsilon }}_{0}\;(/s)$	8.33 × 10^−4^
Room temperature	*T*_0_ (K)	293	Room temperature	*T*_0_ (K)	293
Melting temperature	*T*_m_ (K)	1733	Melting temperature	*T*_m_ (K)	775
Inelastic heat fraction	χ	0.9	Inelastic heat fraction	χ	0.9
Specific heat	*C*p (J/kgK)	441	Specific heat	*C*p (J/kgK)	875
MMC constants	*K* (MPa)	1347	MMC constants	*K* (MPa)	678.7
	*n*	0.425		*n*	0.138
	*c*_1_	0.147		*c*_1_	0.104
	*c*_2_	964.3		*c*_2_	335.6
	$c_{\theta }^{c}$	1.0		$c_{\theta }^{c}$	1.0
	$c_{\theta }^{s}$	1.147		$c_{\theta }^{s}$	1.036
	*D*_4_	−0.036		*D*_4_	0.011
	*D*_5_	26.75		*D*_5_	0.0
	*D*_6_	7.899		*D*_6_	1.0

**Table 5:** Experimental data and numerical simulation results.

5 mm Thick Monolithic 316LASS Target			2024+316L Bilayer Target		
*V*_0_ (m/s)	*V*_i_ (m/s)		*V*_0_ (m/s)	*V*_i_ (m/s)	
	Test	MMC		Test	MMC
287.8	215.4	249.1	146.8	0.0	0.0
265.1	216.4	226.7	209.8	0.0	0.0
167.4	0.0	0.0	246.9	0.0	0.0
219.7	180.1	178.7	258.1	7.38	134.1
183.2	134.1	132.1	268.2	138.48	159.4
205.8	160.8	163.0	253.5	101.82	113.5
153.5	0	0	283.6	148.31	191.2
167.5	92.2	0	289.4	216.34	202.1
352.3	307.1	309.5	322.2	247.97	253.8

**Table 6:** Local and global energy dissipation in targets under different impact velocities.

Target Configuration		Component/Plate	Energy (J)	Percentage (%)	Impact Velocity (m/s)	Projectile Kinetic Energy (J)
Monolithic 316L ASS		*W*_P_^Local^	137.5	34.5	177.6	399.0
		*W*_P_^Global^	45.84	11.5		
		*W*_P_^Local^	157.7	17.6	265.7	893.1
		*W*_P_^Global^	12.6	1.4		
Bilayer target	Front plate	*W*_P_^Local^	98.6	11.0	265.7	893.1
		*W*_P_^Global^	1.43	0.16		
	Rear plate	*W*_P_^Local^	262.8	29.4		
		*W*_P_^Global^	175.8	19.7		

## Data Availability

The original contributions presented in this study are included in the article. Further inquiries can be directed to the corresponding authors.
